# The disappointment of financial support measures during the COVID-19 pandemic among small business managers’ in Sweden

**DOI:** 10.1007/s43546-022-00347-7

**Published:** 2022-10-09

**Authors:** Åsa Tjulin, Stig Vinberg, Bodil J Landstad, Marianne Hedlund, Mikael Nordenmark

**Affiliations:** 1 Department of Health Sciences, Faculty of Human Sciences, Mid Sweden University, Östersund, Sweden; 2 Unit of Research, Education and Development, Östersund Hospital, Östersund, Sweden; 3 Department of Social Work and Health Sciences, Norwegian University of Science and Technology Trondheim, Trondheim, Norway

**Keywords:** Financial support measures, Small business managers, COVID-19, Health, Sweden, I15, I18

## Abstract

The COVID-19 pandemic is viewed as an emergent social phenomenon with several negative effects, e.g., financial decline of small businesses, as well as worsened sense of well-being. The aim of this article is to explore small business managers’ perceptions of governmental financial support measures and relate them to how they experienced their own health and consequences on their work environment. This mixed-method study was performed during the COVID-19 pandemic in Sweden. A survey was conducted during the period from October 2020 to February 2021 and answered by 729 small business managers, followed by ten interviews in March 2021. The key result shows that the managers were dissatisfied with the governmental financial support measures implemented. The results show that the attitudes of the small business managers towards the financial support measures may have had a negative impact on their subjective health. The study indicates a mismatch between the needs of small businesses during the COVID-19 pandemic and how society provides resources through support systems. This in turn may have meant a limitation on the government’s ability to assist small business owners with financial support during the pandemic.

## Introduction

Recent international studies have examined the consequences of the COVID-19 pandemic related to small businesses. The studies view the pandemic as an emergent social phenomenon with several negative effects on small businesses (Blundell and Machin 2020; Shafi et al. 2020; Stephan et al. 2020; Clampit et al. 2021). Small businesses are vulnerable to the challenges of the pandemic due to having fragile economic resources and fewer employees than larger businesses (Kalogiannidis 2020; Stephan et al. 2020 Bartik et al. 2020; Digitally Driven 2021). According to Bartik et al. (2020), small businesses in the tourism, retail, personal services and entertainment sectors in particular have been most affected by the pandemic.

This study contributes to the body of knowledge about small business managers. In this study, small business managers are defined as operating in businesses with fewer than 20 employees in Sweden, either as owner and/or chief executive officer (CEO). The study focuses on how they experienced governmental financial support measures during the COVID-19 pandemic. Around 15 percent of the European labour market is comprised of small business managers (Eurofound 2017). In Sweden around 98 percent of the total number of companies are small businesses (Swedish Agency for Growth Policy Analysis 2019). During the pandemic, around 50 percent of small business managers in Sweden indicated that the profitability of their businesses had deteriorated because of reduced demand for their products and services, supply chain problems and issues in reaching customers (Salesforce Sweden 2021). Several countries have introduced extensive governmental financial support measures such as income protection, expansion of paid sick leave, adjustment support and financial turnover support. In Sweden, governmental support for small businesses included social security contributions, tax deferrals and bank loans for micro- and small-sized businesses, capital injections in strategically important businesses, and support for the start-up of small businesses (Tetlow and  Dalton 2020). The financial support measures were administrated by the Swedish Agency for Economic and Regional Growth. However, studies from UK and Sweden indicate that many small business managers did not apply for any governmental support since they considered that they did not meet the government requirements, or they did not consider themselves eligible for the support (Blundell and Machin 2020; Eib and Berhard-Oettel 2020).

During the early phase of the pandemic, a European study showed that the self-employed (who are often small business managers), reported significantly higher job insecurity and a worse domestic financial situation compared to employees (Eurofound 2020). A similar study in spring 2021 showed that nearly half of this group of managers reported that they were dealing with financial fragility (Eurofound 2021). Hence, an additional consequence of the pandemic may be a negative influence on the well-being of the managers. According to Yue and Cowling (2021), the reduction in work hours and income for self-employed people and small business managers were associated with a deterioration in subjective well-being compared to organizationally employed individuals. This is confirmed by a Swedish study (Eib and Berhard-Oetell 2020), which shows lower scores for well-being among Swedish self-employed people and small business managers compared to scores before the pandemic. Moreover, other recent studies of the Swedish context show that small business managers reported an increased workload and extended work tasks during the pandemic (Vinberg & Danielsson 2021), and the risk of sickness presenteeism was related to business impact, increased work hours and a higher level of work-life conflict (Vinberg et al. 2021).

Battisti and Deakins (2017) claim that businesses need to be proactive and integrate their resources to identify new opportunities when circumstances are unpredictable and uncertain. Other studies show that the emphasis should be on dynamic capabilities such as the ability to transform routines, resources and lessons learnt when the market rapidly shifts, in this case due to the COVID-19 pandemic, (Eisenhardt and Martin 2000; Belitski Guenther Kritikos and Thurik 2021). In addition, it is important to use external sources, for instance networks, to survive and recover from a disaster situation when internal resources are limited (Battisti and Deakins 2017). Since the pandemic has not impacted all small businesses in the same way, due to demographics and sectors, some have increased their profits during the pandemic due to increased demand for their services and products (Bartik et al. 2020; Blundell and Machin 2020; Katare et al. 2021). Katare et al. (2021) show that small businesses adapted their operations in terms of how they interacted with customers, procured supplies and increased media presence. Further, in a Swedish study of small business managers nearly half of the respondents reported they introduced new products or services and new processes, increased their marketing methods and attempted to gain new customers (Vinberg et al. 2021).

The aim of this article is to explore small business managers’ perceptions of governmental financial support measures and relate them to how they experienced their own health and consequences on their work environment. The study was conducted during the COVID-19 pandemic in Sweden.

## Method

### Study design

An explanatory sequence mixed-method design was applied, which entailed quantitative data being analysed first and then explained in more detail using qualitative data (Creswell and Plano Clark 2017). A mixed-method design allows for contextual information about the macro level analysis of the financial support measures to be combined with a micro level analysis about small business managers’ experiences of this support system. This allowed us to analyse the research questions using different data (Creswell and Plano Clark 2017) and confirm quantitative data through the interpretation of qualitative data to identify any complementarity between the data sources (Bryman 2006).

### Quantitative data

A web-based cross-sectional survey was distributed to managers via an email with a link to the survey which was provided by Netigate (https://www.netigate.net). Most of the questions were based on Eurofound’s European Quality of Life Survey (EQLS) and European Working Conditions Survey (EWCS) (Eurofond 2020). The questions aimed to capture the immediate impact of the COVID-19 pandemic on the way people in Europe live and work. The survey was distributed to 796 small business managers during the period October 2020 to February 2021 (Table 1). Different sectors and regions of Sweden were targeted and 729 small business managers with less than 20 employees answered the questionnaire. The distribution of employees in the sample varied from 34% solo business, 47% had 1 – 9 employees in, and 19% had 10–19 employees. A non-probability sample was used representing common Swedish small business sectors and several geographical regions in Sweden.

**Table 1:** Sample questionnaire data – line of business

	Frequency	Percent
Valid		
Agriculture	29	3.6
Industry	43	5.4
Construction	76	9.5
Trade and tourism	111	13.9
Transport	49	6,2
Financial services	55	6.9
Public services	15	1.9
Education	39	4.9
Health	54	6.8
Other services	258	32.4
Total	729	91.6
Missing		
System	67	8.4
Total	796	100.0

The EQLS and EWCS use validated questions and thorough procedures for questionnaire construction, sampling and interviewing when comparing individuals in European countries (Eurofond 2020). Permission has been granted for this present study to use Eurofound’s questions and questionnaire, and the respondents answered electronically and anonymously. The questionnaire consisted of 76 questions divided into four clearly differentiated sections: background information, working conditions, work-life balance and well-being, and questions about the COVID-19 pandemic. There were also open questions about experiences in relation to the pandemic. In this study, we used the questions with fixed-response options related to subjective health outcomes and governmental support measures in the quantitative part of the study, and the open questions in the qualitative part.

#### Analysis of fixed-response options

The quantitative analyses included small business managers with less than 20 employees. The analyses used three variables from the questionnaire that indicate subjective health: “work-related health”, “general health” and “mental health”. “Work-related health” was measured by the following question: "Do you perceive that your work influences your health?”. The response alternatives were 1. Yes, overwhelmingly positively, 2. No and 3. Yes, overwhelmingly negatively. “General health” was measured by the single-item question “In general, how is your health?”. The scale was 1 to 5 (1 = very good, 5 = very bad). This question has been widely used as an epidemiological instrument to predict different health-related outcomes (Lorem et al. 2020).

“Mental health” was measured by the 5-item World Health Organization Well-Being Index (WHO-5) (Topp Dinesen Østergaard Søndergaard and Bech  2015). In the present study, the index is defined as a measure of mental health. It consists of five items about the respondent’s perceptions of how they felt over the preceding two weeks including: cheerful and in good spirits, calm and relaxed, active and vigorous, fresh and rested, and interested in things generally. The responses have been summarised into an additive index varying from 0 to 25 (the higher the value, the poorer the level of mental health), and the calculated Cronbach’s alpha was 0.91.

The following questions were used to analyse which governmental support the managers applied for and whether they received such support since the outbreak of the COVID-19 pandemic: Have you received or requested “Deferral, reduction or cancellation of tax, bills, loans or debt payments”, “Wage support” and “State aid for businesses”. The response alternatives were 1. Have received, 2. Have requested but not yet received, 3. Have requested but the request was rejected, 4. No/Not applicable to me.

In terms of how the managers experienced different governmental support, four questions about the manager’s attitude were used regarding whether support measures were clear and transparent, whether it was easy and efficient to get financial support, whether financial support was fair, and whether the financial support reached those who needed it most. The response alternatives vary from 1 to 4 (1 = agree totally, 4 = not agree).

The quantitative data was analysed by calculating percentages and bivariate correlations (Pearson).

### Qualitative data

#### Open questions from the questionnaire

For the qualitative design and analysis, the following open-answer questions were used: How has your work environment and health been affected by the COVID-19 pandemic? Are there any lessons for policymakers and authorities to learn for future crises? Are there are any lessons to be learnt for your own company regarding COVID-19? After removing the short answers, yes, no and I don’t know, the three questions resulted in 757 open-ended answers that were analysed.

#### Interviews

In addition to the open-ended survey questions, 10 interviews were conducted with small business managers with fewer than 20 employees. Data was collected during the pandemic in March 2021 via telephone or video-link, based on individual preference. The individuals varied in age, gender and industry (Table 2). The interviews lasted for 60 min on average. During seven of the interviews two researchers were present, one as the active interviewer and the other as observer as well as taking field notes and asking clarifying questions if needed. Three interviews were conducted by only one researcher. A semi-structured open-ended interview guide with questions about the interviewee’s background, experience of the pandemic in terms of their business, and experience of governmental financial support, their work environment and the manager’s health during the pandemic. The interviews were recorded and transcribed verbatim by a professional transcriptionist.

**Table 2:** Sample Interviews

Country	Sweden
Managers, total	10
Gender	
Male	4
Female	6
Age	
< 40	1
41–50	2
51–60	3
> 61	4
Education	
High school	3
Vocational training school	2
Upper secondary school	1
University	4
Civil status	
Married/co-habiting	9
Single	1
Industry	
Building & mechanical construction	2
Service industry (Pre-school, wellness services, food industry, media, animal husbandry, sales businesses)	8

#### Qualitative data analysis

The qualitative analysis of both interviews and open-ended questions in the survey was conducted through back-and-forth inductive content analysis (Elo and Kyngäs 2008; Patton 2014). The first step of the analysis was to read and condense meaning using NVivo. This led to descriptive codes for the condensed meanings regarding the governmental financial support measures. The codes related to experiences of support packages, sickness absence, impact on business, information flow, work organisation, dissatisfaction, lessons learnt, finances, impact on their work environment and their own health. After the first step, the authors read and discussed, compared and agreed on the descriptive codes and sought to bring them into a broader analytical framework (Elo and Kyngäs 2008; Patton 2014). This step of the analysis involved interpretative constant comparison with the quantitative data from the survey as well, not only the open-ended question answers, to obtain analytical reflexivity (Srivastava and Hopwood 2009). Three categories emerged: Financial support measures, Consequences of restrictions on health and the work environment, and Need for improvements.

### Methodological considerations

The strengths of using a mixed-method design are that it allows for a combination of data, seeking corroboration between quantitative and qualitative findings, as well as an expansion of how the phenomenon of COVID-19 affected small business managers at a certain point in time during the pandemic. Combining datasets provides a stronger foundation for drawing conclusions about the study results. The quantitative data provides an understanding of the attitudes managers have in relation to governmental economic support measures, and how they rate different well-being outcomes. The qualitative data can enable an increased understanding of how the managers perceive the impact of the pandemic on their businesses and their own health. It also allows an in-depth understanding of how they acted, or not, in relation to governmental support measures, restrictions on society and lessons learnt for the future. However, there are limitations when interpreting the results related to who participated in the interviews, since the small business managers interviewed were not specifically chosen based on industries that are most affected by the COVID-19 pandemic (e.g., tourism, hospitality, restaurants, entertainment and the air transport industry), and data was primarily gathered from a single geographical area. On the other hand, the open-ended questions in the quantitative survey were conducted across industries and regions in Sweden and allowed for triangulation of analysis and trustworthiness. A limitation of the quantitative data is that the sample was not randomly selected, nor could the line of business named “other services” be specified. However, the sample is quite large and covers small business managers in several sectors and regions in Sweden. A further strength is that the questionnaire items have been used in other European investigations and include validated questions.

### Ethical considerations

The study was approved by the Swedish Ethical Review Authority (Dnr 2020–05,223). Informed written consent was sent to all participants of the interviews and questionnaires, and additional verbal consent was obtained at the start of the interviews. Data was treated confidentially. The managers were assured that they could withdraw from the study at any time without needing to give any explanation. All data was properly stored according to the Swedish Act on Ethical Review of Research Involving Humans (SFS 2003:460, 2005).

## Results

The result section merges quantitative and qualitative data to show the results of a comprehensive analysis. Results from the quantitative data analysis are explained in more detail with results from the qualitative analysis.

### Financial support measures

The results from the questionnaire show that relatively few small business managers seem to have applied for and received financial support measures from the government. Table 3 shows that about 45 percent of the small business managers requested deferral, reduction or cancellation of tax, bills, loans or debt payments and only 17 percent of those received the support. The same patterns can be seen regarding wage support and government aid to businesses; around 38 percent requested these types of support, only 10 percent received wage support and 12 percent received government aid to businesses.

**Table 3:** Percentage of small business managers that have received or requested economic support measures since the outbreak of the COVID-19 pandemic

	Have received	Have requested but not received	Have requested but was rejected	No/Not applicable to me	*N*
Deferral, reduction or cancellation of tax, bills, loans or debt payments	17.2	15.4	12.1	55.4	697
Wage support	9.8	13.3	14.1	62.8	697
Government aid to businesses	11.9	9.9	17.1	61.1	697

Note: internal losses, 32 individuals

In response to a question about how helpful the support measures have been for the small business manager’s financial situation in general, 55.7 percent (response alternatives 1 and 2 on a scale from 1 ‘Not at all helpful’ to 5 ‘Extremely helpful’) of the small business managers stated that they have not been helpful, and 21.9 percent (response alternatives 4 and 5) stated that they have been helpful. This indicates that relatively few small business managers perceived the support measures provided by the government as helpful for their financial situation during the COVID-19 pandemic.

The survey data also displays a prevalent dissatisfaction with the government financial support measures available during the pandemic. Table 4 shows that many of the small business managers were generally dissatisfied with various aspects of the measures provided. The results in Table 4 show that 41 per cent fully or partially agreed that the rules for receiving support were clear and transparent. Only 25 per cent believed that government financial support could be obtained easily and efficiently, and 28 per cent agreed that the support measures were fair. On the question of whether the measures reached those who need them most, just over a third (37%) fully or partially agreed that this was the case.

**Table 4:** Small business managers’ attitudes towards economic support measures during the pandemic (percent)

Attitudes towards economic support measures	Strongly agree/Agree	No view/Disagree	*N*
The rules for receiving support are clear and transparent	41.3	58.7	695
Economic support can be obtained easily and efficiently	24.6	76.4	695
The support measures are fair	27.9	72.1	695
Support reached those who need it most	36.5	63.5	695

Note: internal losses, 34 individuals

The findings from both the open-ended questions in the survey and the in-depth interviews confirm the quantitative results that small business managers showed a dissatisfaction with and lack of trust in government financial support measures in general. The frustration expressed by the small business managers can be seen in relation to the type of support measures offered during the pandemic, as well as a recurring sentiment that they do not trust the government or commercial banks. The financial support measures were not perceived as fair for small companies versus the measures provided to larger companies. Overall, the managers expressed that there was a lack of infrastructure provided by the relevant authority, and that the support measures needed improvement. Further, the managers identified a lack of targeted and clear information about the availability of the financial support measures. They mainly searched for information on their own through various media platforms and governmental websites. For some small business managers, the information about financial support was clearer and more targeted if they were connected to an industry organisation or had access to an occupational health service. Thus, the managers strongly suggest that there should be greater understanding of how small companies operate. They believe that targeted types of financial support measures are warranted that match the needs of these companies since they differ to those of larger companies. One interviewee expressed their frustration as follows:

"I’m incredibly disappointed in decision-makers and government authorities. It’s them who have introduced restrictions so that people can’t do their jobs, but they are not compensating the people who really need help. The only thing they’ve offered is loans, which of course have to be repaid, and then with interest. Only the State gains from that. They should make sure they help people properly, without any messy, complex rules – even small business managers with a turnover of less than SEK 180,000. Do they want small businesses to go bankrupt? I actually think they do! They pat themselves on the back and say that they’ve been so good at helping companies. Well yes, large companies! Small business managers – not one bit! It’s a betrayal, that’s what it is when we’ve paid so much tax all these years!" (Sp1).

The managers experienced that the pandemic created different financial circumstances for the company during different periods of the pandemic, especially in the beginning when the uncertainty in the market was hard to predict. However, they did not always apply for support measures despite "difficult times". Instead, they used their own economic buffers. Various explanations to these challenges are revealed by the analysis. One explanation was uncertainty about whether they belonged to the target group for governmental financial support measures. Another explanation was uncertainty due to the unclear rules for those businesses that did not have a union contract agreement, illustrated by one of the managers:

"I’ve had quite a lot of conversations with Tillväxtverket (Swedish Agency for Economic and Regional Growth) and discussions about how we should interpret it all… the rules for people who don’t have a collective agreement through the union. /–-/ And Tillväxtverket was a bit unclear, I called up and spoke to them many times, and they didn’t really know how the rules were to be interpreted either. So you got slightly different answers depending on who you spoke to. /–-/ And this created uncertainty. /–-/ Of course Tillväxtverket must have received loads of questions about these rules, because there are lots of smaller businesses, everything from hairdressers to whatever it might be where they don’t have collective agreements. Collective agreements are a fairly big undertaking, although we have equally good conditions for our employees as collective agreements have, entering into them means a bit more than that.” (Ip1).

The results from both the qualitative and the quantitative results show that small business managers felt that the inequity of financial support measures offered during the pandemic showed that there were shortcomings in the understanding of how small businesses work. They argue that greater consideration needs to be given to the businesses and the resources to which they have access, and that there is a need for transparency in the application process.

### Consequences of restrictions on health and the work environment

The quantitative data contains several indicators of health and well-being. There was general dissatisfaction with how the government support measures have been organised in terms of clarity, equity and opportunities to receive support. As demonstrated in Table 5, dissatisfaction with the way in which the support measures were designed led to negative consequences on the subjective health of the small business owners.

**Table 5:** Correlations between attitudes towards the economic support measures and subjective health. Pearson (*N* = 695)

Attitudes towards economic support measures	Health affected by work	Mental health	General health
The rules for receiving support are clear and transparent	0.097*	0.132***	0.138**
Economic support can be obtained easily and efficiently	0.092*	0.194**	0.149**
The support measures are fair	0.069	0.184**	0.118**
Support reaches those who need it most	0.136**	0.169**	0.114**

^***^P = 0.001 **P = 0.01 *P = 0.05

The results show that the greater the dissatisfaction with the support measures, the higher the risk of experiencing subjective poor health. The question regarding whether the small business managers considered that work has affected their health correlates significantly with all the questions about their experience of the economic support measures, except for the question concerning justice. Thus, the greater the dissatisfaction with the support measures, the higher the risk that work had a negative effect on the health of the small business managers. Both the WHO-5 (indicating mental health) and the question concerning the experiences of general health correlated with all questions regarding experiences linked to the support. This means that the greater the dissatisfaction with the support measures, the worse the mental and general health of the small business managers.

The open-ended questions in the survey show a spectrum of experiences regarding the impact of the pandemic on the small business managers’ work environment and health. Experiences ranged from feeling decreased well-being in the prevailing societal situation due to restrictions, isolation and changed daily routines (e.g., changed exercise patterns), to feeling no impact at all. On the other hand, the findings are coherent in relation to the managers’ views of the survival of their business; they did not intend to give up and remained self-confident that they would be able to push through the pandemic.

In terms of short-term sick leave, the managers experienced an increased level in their companies. Employees complied with the restrictions, e.g., staying home if any symptoms were felt and taking medical tests for COVID-19. Conversely, the increased short-term sick leave did not have a negative impact on business operations or profitability. Some employees continued to work from home and were able to perform their tasks even though they had mild symptoms and waited for test results. Those unable to work from home while on sick leave did not experience an increased workload and a need to catch up when returning to work.

During the interviews it became clear that the small business managers did not experience major challenges in adapting their business operations nor work environment to comply with the public restrictions and recommendations. They searched for and sorted through general information about how to adapt and implement governmental pandemic-related restrictions on their own. They refer to sources of information such as press conferences of the government and other government authorities, monitoring the Swedish Public Health Agency's website, and news reporting in general via different media. In contrast to the vague information about economic support and how to translate that into the business, the information about restrictions was clear:

"Well, actually my colleague took care of that… I’m actually not sure exactly how he looked for information, but we simply googled *covid support* or something like that and took that route [laughs], it wasn’t any harder than that.” (Ip 10)

However, there was a lack of information about how these restrictions and recommendations should be interpreted more specifically, and what they meant for specific industries. Managers needed to use their common sense to adapt, for instance, to comply with the requirements for improved hygiene and cleaning routines. Furthermore, recommendations regarding limiting physical encounters meant that there were fewer opportunities to meet existing customers and to establish new contacts. This led to the creation of new ways of organising work. As an example, managers were positive to the increased number of digital solutions for meetings and communications. Some expressed that it was more efficient to meet digitally and thought that sometimes previous physical meetings were inefficient and welcomed the opportunity to use digital tools to develop their business.

“I hope we can have more digital meetings, they are efficient.” (Sp2)

“Focus more on e-commerce and digital marketing, and the chance to work from home more.” (Sp3)

### Need for improvement

Through the responses, ideas emerged from the small business managers on how the government can support smaller companies in any future pandemics or other critical societal events. The managers were dissatisfied due to the lack of financial support measures that were specifically targeted to small businesses. They perceived that the support systems appeared to be built for larger companies.

Further, the managers expressed that information and communication could be faster, clearer and more accessible to small businesses. It should be easier to apply for support and the time lapse for support should be adjusted to different needs. They expressed that the support given by the authorities should be more elastic, flexible and efficient. The managers would like to see an impact analysis of the different measures for small businesses. They thought this was necessary to be better prepared for future crises and societal challenges.

“So I think we have a pretty big democratic problem that will soon arise, where the gaps have widened a lot. And that’s largely because people haven’t worked together to solve this, but instead it feels like they’ve yelled at each other because they wanted to protect their own boundaries. And what I would actually like, if I could get to wave a magic wand, would be that all those decision-makers would once have run their own businesses, been small business managers for a while. Because I think that would have meant they could have steered things better. (Ip 6).

The quantitative results in Table 4 show that a relatively high number of small business managers applied for support, but it was not granted or received in time. Small business managers learned that building a larger financial buffer and reviewing costs was more important than waiting for support from public authorities. Furthermore, they experienced that being confident and developing their own abilities and flexibility was important. This was a way to build a more solid foundation for unpredictable events and not rely entirely on governmental support.

## Discussion

The aim of this article was to explore small businesses managers’ perceptions of governmental financial support measures and relate them to their experience of their own health and consequences for their work environment. The study was conducted during the COVID-19 pandemic in Sweden. The key result shows that the managers were dissatisfied with the governmental financial support measures implemented. The results also show that the attitudes of the small business managers towards the financial support measures may have had a negative impact on their subjective health. Thus, the result may indicate a delayed negative health impact on small business managers. A recent study from France, also covering several industries, shows that there was a higher level of burn-out among entrepreneurs during the COVID-19 pandemic, with the risk of bankruptcy having the largest effect on mental health (Torrés et al. 2021). The qualitative findings imply that restrictions on society and sick leave had minor consequences on business operations and the work environment. Notably, the small business managers interviewed did not belong to the industries most affected by the pandemic i.e., tourism, retail, personal services and entertainment (Bartik et al. 2020).

In line with previous research in UK and Sweden (Blackburn et al. 2021; Blundell and Machin 2020; Eib and Berhard-Oettel 2020), the main findings from this study show that relatively few small business managers applied for and received financial support measures, and they were dissatisfied with how the government structured the support practices and eligibility. The findings can be interpreted by drawing on structuration theory, which means that they can be understood as an intersection where the structural support system creates *opportunities* or *constraints* for the individual small business managers to act upon the economic support measures offered. Structuration theory can illuminate the interface between individuals’ reflexive actions, i.e., agents (small business managers) and their social practices given the social structure. However, there is a duality between the social practices and the interactions of agents, which are an ongoing negotiation, meaning that social structures are not fixed but can be altered due to the actions taken by agents in reproducing or changing their social roles (Giddens 1984). The discussion displays how the small business managers try to orient themselves and act upon various rules within the financial support systems linked to COVID-19, while at the same time trying to adapt to the system.

When it comes to their perception on financial support measures, the findings reveal that unclear information about criteria for eligibility and the application process for financial support measures was experienced by the managers as a constraint to action. This in turn created frustration, dissatisfaction, and a lack of trust in the government’s ability to handle the financial aid, which in the long run may have a negative impact on subjective health. In an international comparison of financial support measures for business in Canada, France, Germany, Ireland, Japan, New Zealand, Norway, Singapore, Sweden, the take-up of support in Sweden was lower than expected due to the design of the schemes, e.g., the loan schemes were less favourable compared to other countries, and the administrative burden of seeking support was considered too high (Tetlow and Dalton 2020). In our study, the small business managers experienced that the financial support measures offered were not equally distributed and considered there to be a shortage of financial support in society. In other words, the support did not always fulfil its intended purpose, nor provide the right type of support at the right time. Further, the small business managers questioned whether the government was familiar enough with the circumstances of small businesses. Dörr et al. (2021) have studied the impact of the early support policies in Germany, and their results show that there were scarcely screening mechanisms in place for eligibility. This in turn prevented the natural filtering mechanism that would have identified businesses with a weak financial situation. This is in line with conclusions drawn from UK research that shows that weaker firms seemed to benefit more from the support system in the short term, but the businesses which were worst affected by the COVID-19 pandemic were not the ones that benefitted most in the long run. Again, there seems to be a discriminatory effect since the support measures and policies are too general, lacking screening mechanisms which leads to a range of different consequences at different levels of society (Belgihtar Moro and Radic´ 2021; Blackburn et al 2021). This implies a need for change in the government information to ease these constraints and prevent uncertainty about financial aid amongst the small business managers.

Bartik et al. (2020) noted that there are differences in how the pandemic impacts different industries, and the response to how to handle financial support measures will not have an equal effect on all businesses. There may be several reasons why some businesses pull through better than others, from the basic nature of the business to its management capacity. Aside from the constraints, the findings also show that opportunities for action were created in relation to health and the work environment. The managers expressed that the individual’s own personal skills for managing their own health and business were dependent on their self-efficacy, their ability to adapt to a changing market, having their own buffer of financial resources, and being able to transform feelings of uncertainty into a fighting spirit. The findings also show the managers’ adaptability to societal restrictions implemented during COVID-19. Here, the small business managers perceived that there was clear communication and information from the responsible administrative authority regarding public health restrictions. Further, the managers felt secure and competent in how they could adapt their market procedures and work environment routines based on the prevailing restrictions, since the interaction between them and society’s structure for information about restrictions worked properly and left agency i.e., room for manoeuvre, for the managers. Drawing on the concept of dynamic capabilities, real-time and in-time information facilitates the situation-specific adaptability of businesses (Eisenhardt and Martin 2000) and absorptive capacity for managers to alter resources and routines to overcome rapidly changing market conditions as shown in an international comparative study covering Europe, Asia, North America, and Australia (Belitski et al. 2021). Findings from the US suggest that smaller businesses had stronger dynamic capabilities to adapt during the COVID-19 pandemic than larger businesses due to their closer relationships with different stakeholders, e.g., customers and suppliers, and shorter decision paths and relationships with their own employees and day-to-day actions taken (Clampit et al. 2021). Further research has shown that small business managers that stay positive in times of crises can cope with the stressors aligned with uncertainty, strengthen business performance (Grözinger et al. 2021), and take immediate action to prevent long-term negative impact (Thorngren and Williams 2020).

## Conclusions and implications

The study results indicates that there is a mismatch between the needs of small businesses during the COVID-19 pandemic and how society provides resources through financial support systems. The mismatch lies within how the arrangement for obtaining information about, applying for and receiving financial support measures creates a less than optimal interaction between small business managers and the structure, thus creating a barrier to applying for support. The structure of the system can be understood as an obstacle to small businesses' room for manoeuvre to apply for financial support measures. This in turn may have meant a limitation on the government’s ability to assist small business owners with financial support during the pandemic. To promote opportunities for small business managers in the future, the findings show a desire to shift the focus of financial support measures from benefits to large companies to the needs of small businesses. To overcome bureaucratic inertia and increase transparency, information about support measures offered must be enhanced and the support structure for financial aid to small businesses needs to change to be better prepared for future societal crises.

Thus, the policy implications drawn from this study are in line with previous international research looking at support offered to businesses in Canada, France, Germany, Ireland, Japan, New Zealand, Norway, Singapore, Sweden (Tetlow and Dalton 2020) and Lithuania (Lastauskas, 2001). It stresses policy implications regarding screening mechanisms for smaller firms to avoid unequal distribution of support and prevent insolvency gaps among financially weak businesses, which may create a delay to economic recovery (Dörr et al. 2021). Additionally, regional, and sectoral differences in how COVID-19 affects smaller businesses should be screened before decisions on eligibility are made (Belgihtar et al. 2021; Lastauskas 2021). Further research can help unravel how governmental support measures have been implemented, examine the consequences of the measures and their effects on small businesses, including the health- and work environment perspective, and assess how to promote policy development for future economic crises. Such research could also aim to deepen the understanding of the interface between the economic support system and the reflexive agents (small businesses), and how structures can be reformed to facilitate equal distribution of measures during crises. Even though the results point to the structure creating more constraints than opportunities, it may be of interest to focus on those small business managers who experienced the economic support system as helpful, so as to deepen knowledge about what works during economic crises on the meso-intersection level between macro- and micro-economics, and why it worked.

## Data Availability

The datasets generated during and/or analysed during the current study are available from the corresponding author on reasonable request.

## References

[CR1] Bartik AW, Bertrand M, Cullen Z, Glaeser EL, Luca M, Stanton C (2020). The impact of COVID-19 on small business outcomes and expectations. Proc Natl Acad Sci U S A.

[CR2] Battisti M, Deakins D (2017). The relationship between dynamic capabilities, the firm’s resource base and performance in a post-disaster environment. Int Small Bus J.

[CR3] Belghitar Y, Moro A, Radić N (2021). When the rainy day is the worst hurricane ever: the effects of governmental policies on SMEs during COVID-19. Small Bus Econ.

[CR4] Belitski M, Guenther C, Kritikos AS, Thurik R (2021). Economic effects of the COVID-19 pandemic on entrepreneurship and small businesses. Small Bus Econ.

[CR5] Blackburn, R., Machin, S., &Ventura, M. (2021) Covid-19 and the self-employed – 18 months into the crisis. CEP COVID-19 analysis, Paper No.025. London: Centre for Economic Performance and London School of Economics and Political Science.

[CR6] Blundell, J., & Machin, A. (2020) Self-employment in the COVID-19 crisis. CEP COVID-19 analysis, Paper No.003. London: Centre for Economic Performance and London School of Economics and Political Science.

[CR7] Bryman A (2006). Integrating quantitative and qualitative research: how is it done?. Qual Res.

[CR8] Clampit JA, Lorenz MP, Gamble JE, Lee J (2021). Performance stability among small and medium-sized enterprises during COVID-19: A test of the efficacy of dynamic capabilities. Int Small Bus J.

[CR9] Creswell JW, Plano Clark VL (2017). Designing and conducting mixed methods research.

[CR10] Dörr JO, Licht G, Murmann S (2021). Small firms and the COVID-19 insolvency gap. Small Bus Econ.

[CR11] Digitally Driven (2021) European Small businesses find a digital safety net during COVID-19. Report. Connected Commerce. Available via Accessed 15 May 2021

[CR12] Eib C, Berhard-Oettel C (2020). Företagare under och efter COVID-19 [Entrepreneurs during and after COVID-19].

[CR13] Eisenhardt KM, Martin JA (2000). Dynamic capabilities: what are they?. Strateg Manage J.

[CR14] Elo S, Kyngäs H (2008). The qualitative content analysis process. J Adv Nurs.

[CR15] Eurofound,  (2017). Exploring Self-Employment in the European Union.

[CR16] Eurofound,  (2021). Living, working and COVID-19 (Update April 2021): Mental health and trust decline across EU as pandemic enters another year.

[CR17] Eurofound (2020). Living, Working and COVID-19. COVID-19 Series. Luxembourg: Publications of the European Union.

[CR18] Giddens A (1984). The constitution of society: Outline of the theory of structuration.

[CR19] Grözinger AC, Wolff S, Ruf PJ (2021). The power of shared positivity: organizational psychological capital and firm performance during exogenous crises. Small Bus Econ.

[CR20] Kalogiannidis S (2020). Covid Impact on Small Business. Int J Social Sci Econ Invent.

[CR21] Katare., B, Marshall., M.I. & Valdir, C.B.  (2021). Bend or break? Small business survival and strategies during the COVID-19 shock. Int J Disaster Risk Reduct.

[CR22] Lastauskas P (2021). Lockdown, employment adjustment, and financial frictions. Small Bus Econ.

[CR23] Lorem G, Cook S, Leon DA, Emaus N, Schirmer H (2020). Self-reported health as a predictor of mortality: a cohort study of its relation to other health measurements and observation time. Sci Rep.

[CR24] Patton, M. Q. (2014). Qualitative research & evaluation methods: Integrating theory and practice. Sage publications.

[CR25] Shafi M, Liu J, Ren W (2020). Impact of COVID-19 pandemic on micro, small, and medium-sized enterprises operating in Pakistan. Res Glob.

[CR26] Srivastava P, Hopwood N (2009). A practical iterative framework for qualitative data analysis. Int J Qual Methods.

[CR27] Stephan, U., Zbierowski P., & Hanard P-J. (2020) Entrepreneurship and COVID-19: challenges and opportunities. KBS COVID-19 research impact papers, No. 2. London: King’s Business School.

[CR28] Salesforce Sweden (2021) Hur påverkar coronopandemin Sveriges små och stora företag? (How does the corona pandemic affect Swedish small and medium sized enterprises*?)* Available via https://www.salesforce.com/se/blog/2021/hur-paverkar-coronapandemin-sveriges-sma-och-medelstora-foeretag.html Accessed 15 Apr 2021

[CR29] Swedish Agency for Growth Policy Analysis (2019) Nystartade företag i Sverige 2018. Statistik 2019:04. Östersund: The Swedish Agency for Growth Policy Analysis.

[CR30] Tetlow G, Dalton G (2020). Support for business during the coronavirus crisis: an international comparison.

[CR31] Thorgren S, Williams AT (2020). Staying alive during an unfolding crisis: How SMEs ward off impending disaster. J Bus Ventur Insights.

[CR32] Topp CW, Dinesen Østergaard S, Søndergaard S, Bech P (2015). The WHO-5 well-being index: a systematic review of the literature. Psychother Psychosom.

[CR33] Torrès O, Benzari A, Fisch C (2022). Risk of burnout in French entrepreneurs during the COVID-19 crisis. Small Bus Econ.

[CR34] Vinberg S, Danielsson P (2021). Managers of micro-sized enterprises and COVID-19: impact on business operations, work-life balance and well-being. Int J Circumpolar Health.

[CR35] Vinberg S, Landstad BJ, Tjulin Å, Nordenmark M (2021). Sickness presenteeism among the swedish self-employed during the COVID-19 Pandemic. Front Psychol.

[CR36] Yue W, Cowling M (2021). The Covid-19 lockdown in the United Kingdom and subjective well-being: Have the self-employed suffered more due to hours and income reductions?. Int Small Bus J.

