# Correction: The disappointment of financial support measures during the COVID-19 pandemic among small business managers’ in Sweden

**DOI:** 10.1007/s43546-023-00450-3

**Published:** 2023-02-16

**Authors:** Åsa Tjulin, Stig Vinberg, Bodil J. Landstad, Marianne Hedlund, Mikael Nordenmark

**Affiliations:** 1 Department of Health Sciences, Faculty of Human Sciences, Mid Sweden University, Östersund, Sweden; 2 Unit of Research, Education and Development, Östersund Hospital, Östersund, Sweden; 3 Department of Social Work and Health Sciences, Norwegian University of Science and Technology Trondheim, Trondheim, Norway

**Correction: SN Bus Econ (2022) 2:166** 10.1007/s43546-022-00347-7

In this article the author family and given names have been interchanged:

Tjulin Åsa

Vinberg Stig

Landstad Bodil J

Hedlund Marianne

Nordenmark Mikael

They should be read:

Åsa Tjulin

Stig Vinberg

Bodil J. Landstad

Marianne Hedlund

Mikael Nordenmark

The original article has been corrected.

